# A multicenter investigation of reablement in Norway: a clinical controlled trial

**DOI:** 10.1186/s12877-019-1038-x

**Published:** 2019-01-30

**Authors:** E. Langeland, H. Tuntland, B. Folkestad, O. Førland, F. F. Jacobsen, I. Kjeken

**Affiliations:** 1 Centre for Care Research Western Norway, Western Norway University of Applied Sciences, P.O Box 7030, 5020 Bergen, Norway; 2 Department of Health and Caring Science, Faculty of Health and Social Sciences, Western Norway University of Applied Sciences, P.O Box 7030, 5020 Bergen, Norway; 3 Department of Health and Functioning, Faculty of Health and Social Sciences, Western Norway University of Applied Sciences, P.O Box 7030, 5020 Bergen, Norway; 4 Uni Research Rokkan Centre, Bergen, Norway; 5 Faculty of Health Studies, VID Specialized University, Campus Bergen, Ulriksdal 10, 5009 Bergen, Norway; 6 National Advisory Unit on Rehabilitation in Rheumatology, Diakonhjemmet Hospital, P.O. Box 23, Vinderen, 0319 Oslo, Norway; 7 Department of Occupational Therapy, Prosthetics and Orthotics, Faculty of Health Sciences, Oslo Metropolitan University, P.O. Box 4, St. Olavs plass, 0130 Oslo, Norway

**Keywords:** Activities of daily living, Adults, Clinical controlled trial, Home-care services, Reablement, Rehabilitation

## Abstract

**Background:**

Reablement is an emerging approach in rehabilitation services, but evidence for its efficacy is rather weak and inconsistent. The purpose of the present study is therefore to investigate the health effects of reablement in home-dwelling adults.

**Methods:**

A multicenter, clinical controlled trial involving 47 municipalities in Norway, with assessments at baseline, and after 10 weeks and at 6 and 12 months. The sample consisted of 707 persons that received a 4–10 week reablement program and 121 underwent treatment as usual.

Primary outcomes were activity performance and satisfaction with performance measured by the Canadian Occupational Performance Measure (COPM, 1–10). Secondary outcomes included the Short Physical Performance Measure Battery (SPPB), the European Quality of Life Scale (EQ-5D-5 L), and the Sense of Coherence Questionnaire (SOC). Overall treatment effects were estimated with mixed-model repeated measures analyses.

**Results:**

Significant treatment effects in the rehabilitation group compared with the control group were found in COPM-Performance and COPM-Satisfaction scores at 10 weeks (mean differences between groups (MD), 1.61, 95% confidence interval (CI), 1.13, 2.10 and MD 1.47, CI 0.98, 1.97, respectively), and at 6 months (MD 1.42; CI 0.82,2.02 and MD 1.37; CI 0.77,1.98, respectively). There were also significant treatment effects in the SPPB-subscales for balance and walking after 6 months, in the total SPPB score and in the subscale for sit-to-stand after 12 months. In the EQ-5D-5 L assessment, significant treatment effects were found in the subscales for mobility, and for usual activities and health after 6 months. There was a significant difference in the SOC after six months.

**Conclusion:**

Reablement seems to be a more effective rehabilitation service for persons with functional decline than traditional home-based services after six months. After 12 months, the differences between the groups decreased.

**Trial registration:**

The trial was registered at ClinicalTrials.gov on October 24, 2014, (retrospectively registered) identifier: NCT02273934.

## Background

Globally, the proportion of older persons in the total population is increasing [1]. Combined with an expected shortage of trained personnel, this will present a challenge to the sustainability of the healthcare system in years to come [2]. Hence, several high-income countries have promoted a shift from residential care to home-based care, believing it to be a more effective and financially sustainable approach [3]. Further, a high proportion of older people prefer to age at home and to be active in their everyday life and society. Consequently, reablement (in some countries termed restorative care or home-based rehabilitation) is an emerging approach in rehabilitation services for community-dwelling adults experiencing functional decline [4].

In a recent Cochrane review, reablement was defined as an intervention that is person-centered and goal-directed, time-limited and intensive. Typically, it is provided by a multidisciplinary team in the home setting or in the local community, and focuses on supporting independence. Participants must have an identified need for formal care and support, or be at risk of functional decline [5]. This means that, although reablement consists of general features, the intervention varies in content because of the individual tailoring of goals, length and intensity of the intervention. Moreover, the mix of intervention components, skills-mix and setting of the rehabilitation (home or institution), may vary within and between countries. Hence, studies have emphasized different aspects of the intervention, even if they comply with the criteria from the Cochrane review [5].

The effect of reablement on independence in daily activities has been summarized in six systematic reviews [5–10]. The results of the reviews are inconsistent in terms of whether reablement leads to improved independence. Three reviews found limited improvement in favor of reablement [5, 6, 9], whereas three others were inconclusive [7, 8, 10]. One systematic review and five single studies explored whether reablement improved physical functioning. While the systematic review was inconclusive [10], three single studies were in favor of reablement [11–13], whereas two were not [14, 15]. As such, no firm conclusions on whether reablement improves physical function can be drawn. Three systematic reviews and two studies have examined whether reablement improves health-related quality of life [5, 7, 10, 16, 17]. Although these studies have revealed a trend in favor of reablement, the results concerning quality of life remain inconclusive. Reablement is a holistic, health promoting approach. However, no studies have examined whether reablement improves sense of coherence (the main concept in the health-promoting theory of salutogenesis). Hence, the main purpose of this multicenter, clinical controlled trial was to investigate the effects of reablement in home-dwelling adults on daily activities, physical function, health-related quality of life and coping as a sense of coherence.

## Methods

This was a large multicenter, clinical controlled trial involving 47 Norwegian municipalities, in which the intervention group received reablement and the control group received standard care. The study was commissioned by the Norwegian Directorate of Health. The participants were assessed at baseline, at 10 weeks and at 6 and 12 months. Participants were eligible if they were home-dwelling, over 18 years of age, understood Norwegian, and recently had experienced functional decline. Participants were excluded if they needed institution-based rehabilitation or nursing home placement, or if they were terminally ill or cognitively impaired. The study protocol has been published previously [18]. The study was registered in ClinicalTrials.gov (October 24, 2014, identifier: NCT02273934).

### Recruitment and allocation to groups

The reablement group consisted of 36 municipalities and the control group of eight municipalities. In addition, three municipalities were each divided into two zones where one zone acted as control group and the other as an intervention group. The eight municipalities and three zones that functioned as control groups had the possibility to do so because they had not started to implement reablement, yet. After being referred for reablement, an interdisciplinary team assessed each participant’s need of reablement based on the inclusion criteria.

A local study coordinator in each municipality was responsible for the recruitment and checking inclusion criteria.

### Interventions

#### Reablement

In the Norwegian healthcare context, reablement last for 4–10 weeks. The primary focus is to establish a dialog to identify activities that the individual perceives as being meaningful to improve. The intervention is targeted towards achieving these activity goals. Thus, a patient-specific instrument, the Canadian Occupational Performance Measure (COPM), was used as part of the baseline assessments to provide direction for the modeling of the reablement intervention. Since the COPM includes three occupational performance areas: self-care, productivity, and leisure [19], the intervention might include both physical, cognitive, psychological and social components.

A member of the multidisciplinary reablement team (i.e occupational therapist, physiotherapist, nurse) performed the COPM interview in the participant’s home, which started with the following open question: “What are the most important activities in your life now?” During the COPM assessment, the participant defined up to five activity goals that were essential to her or him. Based on these goals, a rehabilitation plan was developed to promote a match between the activities and goals identified by participants, and professional initiatives. Next, an integrated multidisciplinary team with shared goals collaborated with the participant throughout the whole reablement period. The multidisciplinary team consisted most often of an auxiliary nurse, physiotherapist, occupational therapist, nurse and home helper. Intensive attention was given to encourage participation and stimulate daily training for the participants, including performing their daily tasks themselves. Because individual tailoring is a major principle of reablement, the content of the intervention varied among participants, although the basic features were the same. Further details concerning the content of the intervention can be found in the protocol [18].

#### The control intervention

The control group received standard care. As part of the baseline assessment participants in the control group also underwent the COPM interview, but no rehabilitation plan based on this dialogue was made. In contrast to reablement, standard care was not time-limited, and persisted longer than the ten weeks intervention period if needed. It was delivered according to an administrative decision made by the purchaser unit in the municipality after applications made by the participants. This involved care services such as personal or practical assistance, ‘meals on wheels’, safety alarms, or the provision of assistive technology. It could also involve rehabilitation efforts by health professionals such as occupational therapists, physiotherapists and nurses in the participant’s home or local community. Thus, the standard treatment varied among participants and municipalities.

### Training of the intervention providers and local study coordinator in each municipality

To ensure compliance to the intervention and data collection procedures, we arranged a 2-day course where representatives from all 47 municipalities received training in performing the data collection procedures, as well as in designing and delivering the intervention. On the first day, an expert on the COPM system gave lectures and instructions, including practical exercises. On the second day, the principal investigator and the project coworker presented the data collection procedures and the required key elements of the reablement intervention. Each municipality had a local study coordinator who was responsible for the different procedures employed in the project, including data collection. Each local study coordinator received a training manual, including all the procedures and data collection instruments. They were encouraged to use videos to demonstrate how to perform the COPM interviews and physical function tests. In addition, individual supervision was provided by telephone during the intervention and data collection period, and the local study coordinator and healthcare providers were encouraged to contact the principal investigator if they needed to discuss different issues related to the project.

### Data collection

The participants reported the following sociodemographic characteristics: age, gender, marital status, educational level, and whether they lived alone. They also reported their major health challenge, and other health challenges and they scored their motivation for rehabilitation on a 1–10-point scale, where 10 represented the highest motivation. Given the holistic approach in reablement, we included four instruments that had the potential to capture various effects of the intervention. In addition the local project coordinator in each municipality reported how long the reablement intervention period lasted (weeks) and the intensity of the training (more than two times a day = 1, two times a day = 2, daily = 3, three to four times a week = 4, one to twice a week = 5 or more seldom than once a week = 6).

#### Primary outcomes

The COPM approach was used to measure the participants’ performance of daily activities and satisfaction with that performance. This instrument measures a person’s self-perception of activity performance within three occupational performance areas: self-care, productivity, and leisure [19]. During a semi-structured interview, participants described which activities they experienced as important but difficult to perform. The importance of each activity was rated on a 1–10-point scale (10 meaning very important). Next, the participants were asked to prioritize five of the most important activities and thereafter rate their own activity performance (COPM-P) and satisfaction with it (COPM-S) on a scale from 1 to 10 (a higher score reflects better performance and higher satisfaction). Summed scores for the COPM-P and COPM-S, respectively, were calculated by adding the performance or satisfaction scores and dividing these by the number of prioritized activities. The psychometric properties of the COPM have been found to be adequate in a home-dwelling, older population, and the individual minimal important change has been found to be 3.0 and 3.2 points for COPM-P and COPM-S, respectively [20].

#### Secondary outcomes

The Short Physical Performance Measure Battery (SPPB) was applied to measure physical function. The SPPB aims to identify people at risk of functional decline, and is a screening test for mobility [21]. The SPPB comprises the following evaluations: 1) standing balance including side-by-side standing, semi-tandem and tandem standing; 2) a walking test for 4 m at a regular pace; and 3) standing up and sitting down rapidly five times. For each item, the time required was recorded and converted into points (0–4), thereby giving a total score of 0–12 points. The participants’ preferred walking speeds were calculated based on the 4-m walking test. A walking speed > 1.0 m/s was recorded as normal, a speed of 0.6–1.0 m/s was taken as initial disability and a speed of < 0.6 m/s was taken as reflecting frailty [22]. A systematic review concluded that the SPPB has good validity, reliability and responsiveness [23].

The European Quality of Life Scale (EQ-5D-5 L) was used to measure health-related quality of life. The EQ-5D-5 L comprises a questionnaire and a visual analog scale (VAS). The questionnaire has five domains (mobility, self-care, usual activities, pain/discomfort, and anxiety/depression) [24] which are scored on an ordinal scale from 1 to 5, where a score of 1 is best. Hence, a decrease in score represents an improvement. The ‘health today’ VAS is an indication of how individuals value their current health on a scale of 0–100, with 100 being the best. A structured review of the psychometric properties of the EQ-5D-5 L concluded that there is good evidence for its reliability, validity and responsiveness among older adults [25].

The Sense of Coherence Questionnaire (SOC-13) was used to measure coping related to experiences of coherence (comprehensibility, manageability and meaning) in life. The SOC-13, which was developed by Antonovsky [26], is self-reported and comprises 13 items related to comprehensibility (five items), manageability (four items) and meaning (four items). The total score ranges from 13 to 91, with higher scores indicating a stronger sense of coherence. A systematic review concluded that the SOC-13 appears to be a reliable, valid and cross-culturally applicable instrument for measuring how people manage stress and stay well [27].

The questionnaires were self-reported by participants, usually with health professionals present in case they needed guidance.

### Statistical analysis

We calculated the required sample size based on an earlier study performed on older adults, in which the standard deviation for the primary outcome was shown to be 1.4 for COPM performance and 1.6 for COPM satisfaction [28]. Because this trial was a multicenter study with 47 participating municipalities, we expected that variation in the COPM scores would be larger, so we employed a conservative estimate of 2.5 for the standard deviation. Furthermore, the allocation of participants to the intervention or control group was not randomized, and we estimated that the number of participants in the intervention group would probably be three to four times that in the control group. We aimed to detect a change of one point as statistically significant at a two-sided 5% level with a power of 80%. Based on these estimates, sample size calculations indicated that we needed to include 70 participants in the control group and 260 in the intervention group. Thus, considering the possibility of a relatively high dropout rate (up to 35%) because of frailty among the participants, we calculated that a minimum of 107 and 400 participants were needed in the control and intervention groups, respectively.

Descriptive statistics of the sample’s sociodemographic and clinical characteristics are reported as the mean (± standard deviation) and median values (interquartile range), or as numbers and percentages. Differences at baseline between participants in the two groups were analyzed by independent-sample *t* tests for means and χ^2^ tests for proportions. Probability values are reported two-sided and were considered significant at *p* ≤ 0.05.

The effect analyses are based on the intention-to-treat principle. Such data are suitable for multilevel or hierarchical modeling. Individuals were nested within municipalities, and municipalities were treated as fixed effects when mixed-effect models were applied [29]. To evaluate whether the effect of the intervention varied according to home municipality, linear mixed-effect models were used. Given the multilevel structure of the data (individuals over time within municipalities), we aimed to control for stable differences between municipalities using a so-called fixed-effects model. By employing mixed-effect models we thereby control both the effect of variations between the municipalities as well as the effect of belonging to either the control or reablement group.

In these analyses, the differences between the scores at 10-week, 6-month and 12-month follow-ups and baseline scores were used as dependent variables, and individuals were nested within municipalities in a hierarchical structure. We grouped the participants within their municipalities to assess the effect of locality. Because a baseline score might predict changes in that score, we also included baseline scores for the dependent variables as an independent variable. The statistical analyses were performed using Stata software (version 14.2; StataCorp, College Station, TX, USA).

## Results

### The intervention

It was emphasized that the duration of the reablement period was individually tailored. However, the majority of participants received a reablement intervention for between four and six weeks with a mean of 5.7 weeks. The most frequently reported intensity in the municipalities was training five times a week (48%, *n* = 17) and three to four times a week (33%, *n* = 12).

### Participant flow and study sample

Approximately 17% of the Norwegian population lived in the municipalities included in this study. Both rural and urban municipalities of various sizes were included, from the north to the south of Norway.

**Fig. 1:**
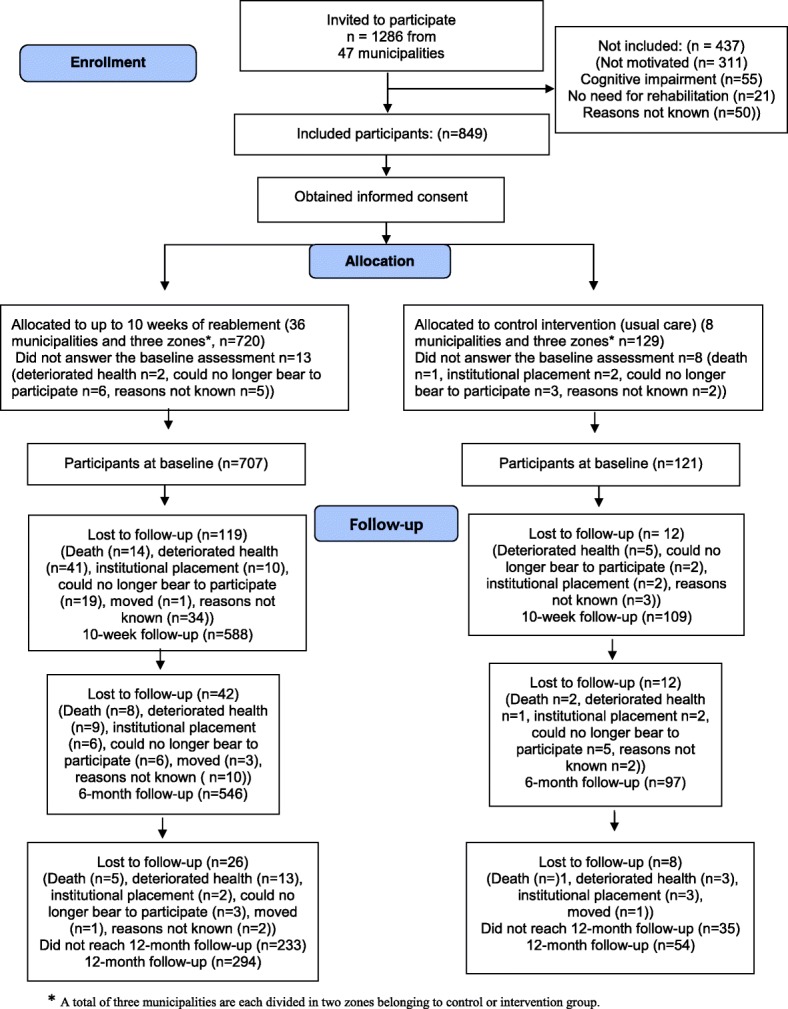
Flow chart of the trial profile

The predefined recruitment period started in April 2014 and ended in June 2015, whereas all data collection ended in December 2015. Of 1286 potential participants, 849 were included in the study. A total of 828 persons answered at baseline: 707 in the intervention group and 121 in the control group. The flow diagram of the study is outlined in Fig. 1.

Due to the predefined termination of data collection in December 2015 a total of 268 (233 in the intervention group and 35 in the control group) did not reach the 12-month follow up, and are categorized as ‘did not reach 12-months follow-up’ in Fig. 1. Dropout rates at the 10-week and 6- and 12-month follow-up times were 13.6, 20 and 26%, respectively.

There were no differences in baseline scores for COPM performance and satisfaction between those who dropped out and those who responded at the 10-week follow up (*p* = 0.65, and *p* = 0.92, respectively).

Table 1 displays the baseline participant characteristics in total and for each group. The participants were on average 78 years old, two-thirds were women, one in five had higher education and two-thirds were living alone. The two groups were similar, except for the motivation variables, where the participants in the reablement group were slightly more motivated than were those in the control group (Table 1).

**Table 1:** Baseline characteristics of participants allocated to reablement group or control group

	All participants (*N* = 828)	Reablement group (*n* = 707)	Control group (*n* = 121)	*p* ^a^
Age, years, mean (SD)	78.4 (10.9)	78.2(11.2)	79.5(9.3)	0.22
Female, *n* (%)	575 (69.3)	488 (68.7)	87 (72.5)	0.41
Higher education, *n* (%)	167 (20.3)	140 (19.9)	27 (22.5)	0.52
Living alone	596 (71.6)	502 (70.7)	94 (78.3)	0.38
Motivation, scale 1–10, (10 is highest), mean (SD)	8.1 (2.1)	8.2 (2.0)	7.4 (2.6)	< 0.001
COPM^b^-P^c^, scale 1–10, (10 is highest), mean (SD)	3.54 (1.62)	3.46 (1.61)	4.00 (1.91)	0.01
COPM^b^-S^d^, scale 1–10, (10 is highest), mean (SD)	3.41 (1.82)	3.31 (1.81)	4.14 (2.1)	< 0.001

^a^Difference between groups (independent-sample *t* tests for means and χ^2^ tests for proportions). *SD* standard deviation of the mean

^b^Canadian Occupational Performance Measure, ^c^Activity performance, ^d^ Satisfaction with performance

They had a range of health challenges, with the most common being fractures (20.3%), dizziness (15.1%), pain (9.9%), stroke (9.1%), arthritis (7.5%) and heart disease (5.9%). In addition, most of the participants reported two other health challenges. For further details, see Table 2.

**Table 2:** Major health challenge

	The whole sample (*N* = 828)		Intervention-group (*n* = 707)		Control-group (*n* = 121)	
Major health challenge	*n*	%	*n*	%	*n*	%
Fracture	168	20.3	149	20.9	19	15.7
Dizziness/balance problems	125	15.1	113	15.9	12	9.9
Pain	82	9.9	70	9.8	12	9.9
Stroke	75	9.1	66	9.3	9	7.4
Arthritis	62	7.5	47	6.6	15	12.4
Heart disease	49	5.9	37	5.2	12	9.9
Orthopedic disease	37	4.5	29	4.1	8	6.6
Neurological disease other than stroke	32	3.9	25	3.5	7	5.8
Pulmonary disease	31	3.7	29	4.1	2	1.7
Back disease/problem	20	2.4	17	2.4	3	2.5
Movement challenges/functional decline	20	2.4	16	2.3	4	3.3
Sight problems/eye disease	19	2.3	13	1.8	6	5.0
Cancer	19	2.3	16	2.3	3	2.5
Mental health problems	13	1.6	13	1.8	–	–
Amputation(s)	12	1.5	11	1.5	1	0.8
Diabetes	11	1.3	10	1.4	1	0.8
Digestion disease	7	0.8	7	1.0	–	–
Urinary infection	7	0.8	5	0.7	2	1.7
Sleep disease/problems	3	0.4	3	0.4	–	–
Other health challenges^a^	36	4.5	33	4.6	3	3.3
Number of additional health conditions mean (SD), min-max	2 (2.06), 0–11		2 (2.04), 0–11		2 (2.17), 0–9	

^a^Health challenges that did not fit into the predefined categories

### Effects for participants

#### Primary outcomes

**Fig. 2:**
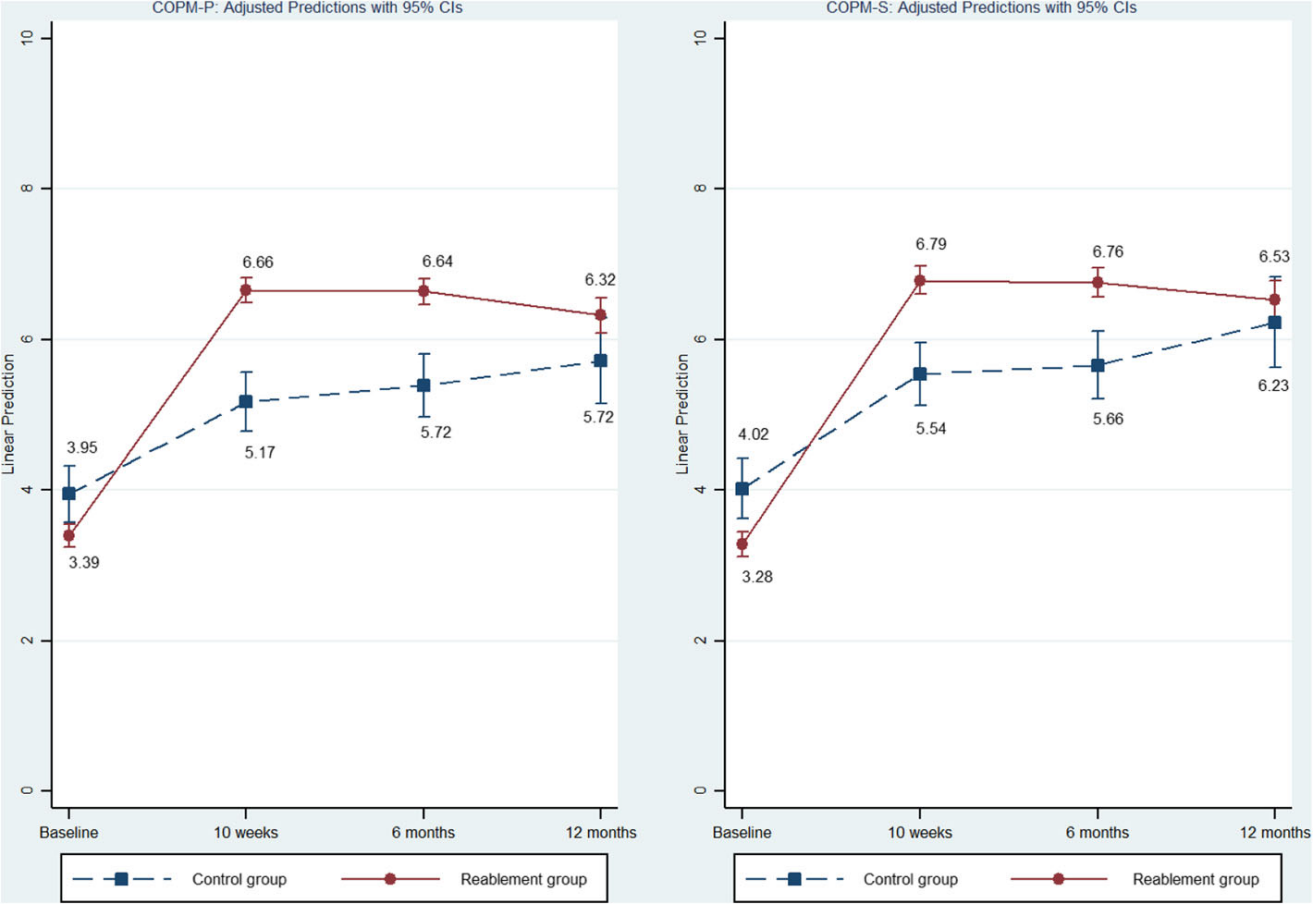
Changes in scores from the COPM-S and COPM-P tests in the control and reablement groups over time

There were significant treatment effects in favor of the reablement group in the primary outcomes activity performance (COPM-P) and satisfaction with performance (COPM-S) at 10 weeks (mean COPM-P and COPM-S differences between groups were 1.61, 95% confidence interval [CI], 1.13, 2.10 and 1.47; 95% CI, 0.98, 1.97, respectively) and at the 6-month follow-up (mean differences between groups, 1.42, 95% CI, 0.82, 2.02 and 1.37; 95% CI, 0.77, 1.98, respectively). At the 12-month follow-up, there was no significant difference in COPM-P scores (mean difference between groups 0.81; 95% CI, − 0.04, 1.66) and in COPM-S scores (mean difference between groups 0.63; 95% CI, − 0.26, 1.51; see Table 3 and Fig. 2).

**Table 3:** Mean (95% confidence interval (CI)) for treatment effects, estimated by a mixed-model linear repeated measures analysis^a^

	Reablement group Mean (95% CI)	*n*	Control group Mean (95% CI)	*n*	Treatment effect Mean (95% CI)	*p-value*
*Primary outcomes*						
Activity performance COPM^b^-P (1–10, 10 is high)						
Baseline	3.46 (3.24, 3.68)	707	4.00 (3.61, 4.40)	120		0.01
10 weeks	3.19 (2.98, 3.40)	588	1.57 (1.12, 2.02)	107	1.61 (1.13, 2.10)	< 0.001
6 months	3.19 (2.91, 3.46)	546	1.77 (1.21, 2.33)	97	1.42 (0.82, 2.02)	< 0.001
12 months	2.76 (2.38, 3.15)	294	1.95 (1.17, 2.74)	52	0.81 (−0.04, 1.66)	0.06
Satisfaction with performance COPM^b^-S (1–10, 10 is high)						
Baseline	3.31 (3.07, 3.54)	705	4.14 (3.71, 4.57)	120		< 0.001
10 weeks	3.43 (3.23, 3.64)	585	1.96 (1.50, 2.42)	107	1.47 (0.98, 1.97)	< 0.001
6 months	3.41 (3.15, 3.67)	543	2.04 (1.48, 2.61)	95	1.37 (0.77, 1.98)	< 0.001
12 months	3.14 (2.74, 3.54)	294	2.51 (1.70, 3.32)	52	0.63 (− 0.26, 1.51)	0.16
*Secondary outcomes*						
SPPB^c^ Total score (0–12, 12 is high)						
Baseline	4.83 (4.50, 5.15)	697	5.61 (4.99, 6.23)	121		0.02
10 weeks	1.72 (1.48, 1.96)	574	0.39 (− 0.10, 0.88)	109	1.33 (0.80, 1.86)	< 0.001
6 months	1.65 (1.39, 1.91)	530	0.42 (− 0.13, 0.96)	96	1.23 (0.65, 1.82)	< 0.001
12 months	1.46 (1.08, 1.84)	278	0.42 (− 0.34, 1.17)	52	1.03 (0.19, 1.86)	0.02
SPPB Balance (0–4, 4 is best)						
Baseline	2.25 (2.11, 2.38)	697	2.39 (2.11, 2.67)	121		0.34
10 weeks	0.47 (0.35, 0.59)	573	0.13 (−0.11,0.37)	109	0.34 (0.09, 0.59)	0.01
6 months	0.42 (0.31, 0.53)	529	0.05 (− 0.18, 0.29)	96	0.36 (0.11, 0.62)	0.01
12 months	0.27 (0.13, 0.41)	278	0.10 (−0.21, 0.40)	52	0.17 (−0.16, 0.51)	0.31
SPPB Walking (0–4, 4 is best)						
Baseline	1.80 (1.67, 1.94)	697	2.12 (1.87, 2.37)	121		0.02
10 weeks	0.56 (0.45, 0.67)	570	0.19 (−0.02, 0.40)	109	0.37 (0.15, 0.59)	< 0.001
6 Months	0.54 (0.40, 0.67)	529	0.17 (−0.08,0.43)	96	0.36 (0.09, 0.63)	0.01
12 Months	0.46 (0.32, 0.61)	278	0.31 (0.01, 0.62)	51	0.15 (−0.18, 0.48)	0.38
SPPB Sit-to-stand (0–4, 4 is best)						
Baseline	0.79 (0.69, 0.89)	696	1.05 (0.84, 1.27)	121		0.03
10 weeks	0.72 (0.63, 0.81)	569	0.11 (−0.09, 0.31)	109	0.61 (0.39, 0.83)	< 0.001
6 Months	0.71 (0.61, 0.80)	528	0.25 (0.02, 0.47)	96	0.46 (0.21, 0.70)	< 0.001
12 Months	0.69 (0.53, 0.85)	278	0.20 (−0.15, 0.55)	51	0.49 (0.11, 0.87)	0.01
EQ-5D-5 L Mobility (1–5, 1 is best)						
Baseline	2.84 (2.75, 2.94)	696	2.65 (2.45, 2.85)	121		0.08
10 weeks	−0.61 (− 0.68, − 0.54)	579	− 0.12 (− 0.29, 0.05)	103	−0.49 (− 0.68, − 0.31)	< 0.001
6 months	−0.57 (− 0.64, − 0.49)	542	−0.20 (− 0.38, − 0.01)	97	−0.37 (− 0.57, − 0.17)	< 0.001
12 months	−0.48 (− 0.59, − 0.36)	288	−0.27 (− 0.54, − 0.01)	54	0.20 (− 0.49, 0.08)	0.17
EQ-5D-5 L Personal care (1–5, 1 is best)						
Baseline	2.04 (1.93, 2.15)	697	1.83 (1.62, 2.04)	121		0.07
10 weeks	−0.48 (− 0.54, − 0.42)	581	− 0.12 (− 0.26, 0.02)	103	−0.36 (−-0.51, − 0.21)	< 0.001
6 months	−0.40 (− 0.47, − 0.32)	543	−0.14 (− 0.31, 0.03)	97	−0.26 (− 0.44, − 0.07)	0.01
12 months	−0.42 (− 0.53, − 0.31)	289	−0.18 (− 0.41, 0.05)	54	−0.24 (− 0.49, − 0.01)	0.06
EQ-5D-5 L Usual activities (1–5, 1 is best)						
Baseline	2.87 (2.76. 2.98)	696	2.74 (2.52, 2.96)	121		0.28
10 weeks	−0.57 (− 0.73, − 0.42)	576	−0.26 (− 0.62, 0.09)	103	−0.31 (− 0.70, 0.08)	0.12
6 months	− 0.64 (− 0.72, − 0.54)	540	−0.34 (− 0.54, − 0.14)	97	−0.30 (− 0.52, − 0.08)	0.01
12 months	−0.64 (− 0.77, − 0.52)	290	−0.38 (− 0.66, − 0.11)	54	−0.26 (− 0.56, 0.04)	0.09
EQ-5D-5 L Pain/discomfort (1–5, 1 is best)						
Baseline	2.65 (2.53. 2.76)	693	2.66 (2.44, 2.89)	121		0.87
10 weeks	−0.21 (−0.28, − 0.13)	577	− 0.87 (− 0.36, 0.01)	103	−0.02 (− 0.21, 0.17)	0.82
6 months	− 0.24 (− 0.33, − 0.15)	537	−0.13 (− 0.33. 0.06)	97	−0.11 (− 0.32, 0.11)	0.33
12 months	− 0.23 (− 0.35, − 0.12)	54	−0.21 (− 0.47, 0.05)	287	− 0.03 (− 0.31, 0.26)	0.85
EQ-5D-5 L Anxiety/depression (1–5, 1 is best)						
Baseline	1.84 (1.75. 1.93)	692	1.68 (1.49, 1.86)	121		0.11
10 weeks	−0.13 (−0.19, − 0.06)	573	−0.24 (− 0.39, − 0.10)	102	0.12 (− 0.03, 0.27)	0.12
6 months	−0.16 (− 0.23, − 0.10)	528	−0.10 (− 0.26, 0.05)	95	−0.06 (− 0.32, 0.11)	0.48
12 months	− 0.19 (− 0.28, − 0.10)	282	−0.20 (− 0.39, 0.00)	54	0.00 (− 0.21, 0.22)	0.98
EQ-5D-5 L Health today (0–100, 100 is high)						
Baseline	49.85 (48.02, 51.68)	687	53.06 (49.16, 56.95)	121		0.13
10 weeks	8.36 (6.89. 9.83)	575	2.28 (−1.07, 5.62)	103	6.08 (2.44, 9.72)	< 0.001
6 months	9.14 (7.03, 11.25)	528	1.52 (−2.86, 5.90)	97	7.62 (2.87, 12.37)	< 0.001
12 months	7.55 (5.33, 9.78)	282	4.79 (−0.301, 9.88)	54	2.76 (−2.80, 8.32)	0.33
SOC-13 Sense of coherence (13–91, 91 is high)						
Baseline	68.45 (67.34, 69.56)	658	69.45 (67.13, 71,76)	118		0.44
10 weeks	0.31 (−0.76, 1.38)	539	−1.41 (−3.56, 0.73)	103	1.73 (−0.56, 4.01)	0.14
6 months	0.29 (−1.07, 1.66)	496	−2.62 (−5.29, 0.04)	94	2.92 (0.08, 5.75)	0.04
12 months	0.71 (−1.04, 2.47)	256	0.02 (−3.38, 3.43)	51	0.69 (−3.02, 4.40)	0.72

^a^Adjusted for the baseline mean value of each variable

^b^COPM:Canadian Occupational Performance Measure

^c^SPPB: Short Physical Performance Measure Battery

^d^EQ-5D-5 L: European Quality of Life Scale

In addition, the intraclass-correlation coefficient suggested that on average, 6% of the variation in the COPM scores was explained by municipality (varying between 12 and 2% at different measurement points for COPM-P and COPM-S; data not shown).

Furthermore, the results demonstrated that those who scored lower on COPM at baseline had significantly better improvement in their COPM scores at all measurement points compared with those with higher baseline scores (*p* ≤ 0.001 at all follow-ups). Regarding the COPM-S, the baseline scores and changes in scores at the second measurement were strongly correlated in both the intervention (*r* = − 0.42) and control group (*r* = − 0.53), with lower scores at baseline correlating with an increase at the second measurement.

Figure 2 shows the development in the main outcomes for COPM-S and COPM-P in the intervention and control group for the different measurement periods. The intervention group had significantly lower scores at baseline than the control group for both COPM-S and COPM-P, (*p* ≤ 0.001) see Table 3). After 10 weeks, 6 and 12 months, there were increases for the intervention group that surpassed the mean scores for the control group. At 12 months, the differences in scores between the control and intervention groups were decreasing, and they were no longer significant.

### Secondary outcomes

#### Physical function (SPPB)

There was a significant treatment effect in favor of the reablement group in the total physical function score and in the subscale sit-to-stand measure after 12 months. In the subscales for balance and walking, there were significant differences after 10 weeks and 6 months, but not after 12 months (Table 3).

#### Health-related quality of life (EQ-5D-5 L)

There was a significant treatment effect in favor of the reablement group in health-related quality of life in terms of mobility, personal care and usual activities and current health after 6 months. Regarding the pain/discomfort and anxiety/depression dimensions, there were no significant differences between the groups at any time point (Table 3).

#### Sense of coherence (SOC-13)

Regarding the evaluation of SOC there were no significant differences between the reablement and control groups at any measurement point, except for SOC at six months (Table 3).

## Discussion

To our knowledge, this was the largest multicenter study of the effects of reablement ever undertaken, including numerous participants in a geographically larger Norwegian population. The main results indicated that reablement significantly improved self-perceived activity performance and satisfaction with performance at 10 weeks (the mean differences in treatment effects between groups in the COPM-P and COPM-S evaluations were 1.61 and 1.44, respectively, *p* ≤ 0.001 for both). Importantly, these significant improvements were maintained at 6 months (mean differences between groups, 1.42 and 1.37, respectively, *p* ≤ 0.001 for both).

As for the secondary outcomes, there were significant improvements in the reablement group for mobility, personal care, current health, physical function, balance, walking and sit-to-stand measures at both 10 weeks and 6 months, but little or no significant changes in the scores for usual activities, pain/discomfort, anxiety or sense of coherence at the same time points. At 12 months, significant differences were found in total physical function and sit-to-stand scores. Thus, the results demonstrate that reablement resulted in both short- and long-term goal attainment (as measured by the COPM), as well as improvements in a variety of other aspects of health and physical function.

These findings are in line with the three reviews that found some evidence for improvement in favor of reablement regarding daily activities [5, 6, 9]. However, in this large multicenter study, significant effects were found after 6 months thereby strengthening the evidence for the positive effect of reablement on daily activities. For physical functioning, the results in our study are promising over a 12-month perspective, and therefore support other studies revealing similar effects [11–13]. Regarding health-related quality of life, our findings suggest that reablement might improve this outcome after 6 months. Therefore, our study supports and confirms the five studies showing that reablement can improve health-related quality of life [5, 7, 10, 16, 17]. These comparisons should, however, be interpreted with caution, since the content, length and intensity in reablement varies between studies.

This study is the first to investigate the effects of reablement on participants’ sense of coherence. However, sense of coherence was only significantly improved at six months. One possible reason for this may be that the participants reported mainly on physical health challenges, whereas previous research has revealed that sense of coherence is particularly linked to mental health [27].

Persons with low initial values in the main outcome measures made the most progress, indicating that those with the lowest initial scores had the greatest potential for improvement. However, we cannot exclude that the negative correlation (*r* = − 0.42) between baseline scores and the changes in scores in the post-intervention measurements reflects a regression-to-the-mean effect [30]. This is supported by our finding that a negative correlation (*r* = − 0.53) was also present in the control group.

Both groups reported relatively high motivation. This is not surprising since low motivation was reported as a main cause for not participating in the study. In addition, previous research has shown that distinct personalized reablement goals create and promote motivation [31, 32]. However, at baseline, participants in the reablement group reported significant higher motivation for reablement than participants in the control group. Since the current study is not a randomized trial, this difference may also be indicative of other important differences between the groups. The effect of reablement should therefore be further explored in larger, randomized controlled trials.

In this trial, the participants’ prioritized activities were used as a basis for developing reablement goals, thereby enhancing communication and encouraging the participants to take an active role to in the rehabilitation process. Furthermore, applying a person-centered instrument such as the COPM might promote participation and motivation [33].

The latter point is supported by our finding that the control group also improved in activity performance and satisfaction with performance in the follow-up periods. The same phenomenon has been reported in previous studies, where the authors suggest that the baseline COPM interview and scoring process might have a therapeutic effect by promoting consciousness and motivating participants to seek solutions themselves, thereby diminishing potential differences between the control and intervention groups [34, 35]. However, the control intervention was not time-limited and could persist longer than the ten weeks intervention period in reablement. This may be one reason why the differences between groups diminished over time. Further, it is reasonable to think that one reason for the significant difference between the groups at 10 weeks follow-up is due to faster goal-attainment in the reablement group. This might indicate that reablement makes people better quicker, but do not have better effect than traditional services in the longer run. However, we need more follow-up studies to conclude on the long term effects of reablement.

Our multilevel analysis showed that there was little variability between municipalities, and that the change occurred mainly on the individual level, regardless of locality (data not shown). This suggests that the motivation to improve performance of different daily activities and work with oneself comes from within, stimulated and supported by health professionals’ competence.

Optimizing capacities allows each person to make the best of their resources, despite functional limitations. It is a newly developed concept for reablement that purports to explain how various strategies can be used to optimize the functions of older adults to enable them to age in place [36]. The identified strategies are: accepting the motivational work of the healthcare providers and the reablement service offered; training in physical fitness and everyday life activities to increase physical capacities; adapting to the environment; and building confidence based on rehearsals of activities and exercises, increased knowledge and support from others [36]. Together, these strategies may lead to optimal functioning as stated by reablement theory, enabling participants to manage as well as possible in their own homes and local communities.

One of the prime reasons for governments’ interest in reablement is to manage health and social care costs resulting from population aging. The cost-effectiveness of reablement is therefore currently investigated in another paper, where analyses are based on data from the present multicenter study.

This study had some methodological challenges. One was that a possible lack of compliance with the intervention and data collection procedures comprised a possible threat to the reliability of the study. Monitoring compliance was difficult because of the large number of municipalities and healthcare professionals involved in the trial, including professionals leaving the study. A crucial part of reablement is the quality of the COPM assessment. Communication skills such as empathy, listening and ability to let the participant be the expert on his or her life situation is a prerequisite for the possibility of participants to set their own goals [37]. The reablement intervention was also tailored individually, which further increased the complexity for analysis. However, several measures were performed to secure compliance and treatment fidelity. Healthcare professionals in all municipalities had received training in the study procedures including the COPM interview and the content of the intervention. Furthermore, if a professional left the study, their replacement received sufficient training in both the intervention and data collection procedures. In addition, the principal investigator had regular contact with each municipality to ensure compliance with the procedures, and checked all incoming data material continuously to detect and correct any misunderstandings or missing values.

However, due to determination of the inclusion period, not all participants reached the follow-up at 12 months. This makes the results from this time point less trustworthy because of the lack of statistical power. In addition, the intervention group reported significantly lower values in the primary outcomes for the COPM-P and COPM-S tools than did the control group at baseline. This means that we cannot exclude that there has been a recruitment bias. The inclusion criteria were dependent on a clinical judgment that may have been practiced different between municipalities However, we controlled for these differences in the statistical analysis. The study was also limited by dropouts during the trial period, with a 26% drop-out rate at 12 months. However, there were no significant differences at baseline in the primary outcomes between those who dropped out and those who responded at 10 week follow up. In addition, a mixed model analysis is robust concerning missing values, because data at all time points are used, even if participants are missing at one of them [29].

Another potential problem that may have produced an ascertainment bias is that the COPM was used both to guide the intervention treatment and to measure outcome. However, the participants had not access to their previous scoring at the follow-ups.

An important strength of the trial was that it occurred in a natural setting, so its practicality, feasibility and - to some extent - generalizability may be high. That participants comprised a heterogeneous group from different parts of Norway also strengthened the generalizability of the results.

In this trial, the allocation of participants to the reablement group or control group was not randomized. However, it is an advantage that the professionals working with reablement or usual care were working in different municipalities or zones, and thus contamination from one arm of the study to the other was unlikely.

The practicing of exclusion criteria such as cognitively impairment was based on the health professionals’ clinical judgment. This means that people with a lower degree of cognitive impairment may have participated. However, since COPM was a mandatory part of the baseline assessments, participants had to be able to complete the COPM interview and scoring, a process that require relatively high cognitive function.The inclusion criteria regarding age was 18 years or older, but the actual recruitment gave a sample with a mean age of 78 years. This shows that reablement in Norway is currently mainly offered to older people, even though it might be suitable for younger ones.

## Conclusion

This multicenter, clinical controlled trial demonstrated that reablement had significant effects on activity performance, satisfaction with performance and many other health outcomes after 6 months. After 12 months, the positive effects decreased. This study makes an important contribution to our knowledge of rehabilitation approaches for community-dwelling older adults.

## Abbreviations

COPM: Canadian Occupational Performance Measure
EQ-5D: European Quality of Life Scale with five dimensions
HRQoL: health-related quality of life
SOC: Sense of Coherence questionnaire
SPPB: Short Physical Performance Battery
VAS: Visual analog scale
